# Identifying of immune‐associated genes for assessing the obesity‐associated risk to the offspring in maternal obesity: A bioinformatics and machine learning

**DOI:** 10.1111/cns.14700

**Published:** 2024-03-27

**Authors:** Yanxing Shang, Xueqin Wang, Sixuan Su, Feng Ji, Donghai Shao, Chengwei Duan, Tianpeng Chen, Caixia Liang, Dongmei Zhang, Hongjian Lu

**Affiliations:** ^1^ Medical Research Center, Affiliated Hospital 2 Nantong University Nantong China; ^2^ Jiangsu Provincial Medical Key Discipline (Laboratory) Cultivation Unit, Medical Research Center Nantong First People's Hospital Nantong China; ^3^ Nantong Clinical Medical College of Kangda College of Nanjing Medical University Nantong China; ^4^ Nantong Municipal Key Laboratory of Metabolic Immunology and Disease Microenvironment Nantong First People's Hospital Nantong China; ^5^ Department of Endocrinology, Affiliated Hospital 2 Nantong University Nantong China; ^6^ Department of Pathogen Biology, Medical College Nantong University Nantong China; ^7^ Department of Rehabilitation Medicine, Affiliated Hospital 2 Nantong University Nantong China

**Keywords:** bioinformatics analysis, biomarkers, differentially expressed genes, hypothalamus, obesity

## Abstract

**Background:**

Perinatal exposure to maternal obesity predisposes offspring to develop obesity later in life. Immune dysregulation in the hypothalamus, the brain center governing energy homeostasis, is pivotal in obesity development. This study aimed to identify key candidate genes associated with the risk of offspring obesity in maternal obesity.

**Methods:**

We obtained obesity‐related datasets from the Gene Expression Omnibus (GEO) database. GSE135830 comprises gene expression data from the hypothalamus of mouse offspring in a maternal obesity model induced by a high‐fat diet model (maternal high‐fat diet (mHFD) group and maternal chow (mChow) group), while GSE127056 consists of hypothalamus microarray data from young adult mice with obesity (high‐fat diet (HFD) and Chow groups). We identified differentially expressed genes (DEGs) and module genes using Limma and weighted gene co‐expression network analysis (WGCNA), conducted functional enrichment analysis, and employed a machine learning algorithm (least absolute shrinkage and selection operator (LASSO) regression) to pinpoint candidate hub genes for diagnosing obesity‐associated risk in offspring of maternal obesity. We constructed a nomogram receiver operating characteristic (ROC) curve to evaluate the diagnostic value. Additionally, we analyzed immune cell infiltration to investigate immune cell dysregulation in maternal obesity. Furthermore, we verified the expression of the candidate hub genes both in vivo and in vitro.

**Results:**

The GSE135830 dataset revealed 2868 DEGs between the mHFD offspring and the mChow group and 2627 WGCNA module genes related to maternal obesity. The overlap of DEGs and module genes in the offspring with maternal obesity in GSE135830 primarily enriched in neurodevelopment and immune regulation. In the GSE127056 dataset, 133 DEGs were identified in the hypothalamus of HFD‐induced adult obese individuals. A total of 13 genes intersected between the GSE127056 adult obesity DEGs and the GSE135830 maternal obesity module genes that were primarily enriched in neurodevelopment and the immune response. Following machine learning, two candidate hub genes were chosen for nomogram construction. Diagnostic value evaluation by ROC analysis determined Sytl4 and Kncn2 as hub genes for maternal obesity in the offspring. A gene regulatory network with transcription factor–miRNA interactions was established. Dysregulated immune cells were observed in the hypothalamus of offspring with maternal obesity. Expression of *Sytl4* and *Kncn2* was validated in a mouse model of hypothalamic inflammation and a palmitic acid‐stimulated microglial inflammation model.

**Conclusion:**

Two candidate hub genes (*Sytl4* and *Kcnc2*) were identified and a nomogram was developed to predict obesity risk in offspring with maternal obesity. These findings offer potential diagnostic candidate genes for identifying obesity‐associated risks in the offspring of obese mothers.

## Graphical abstract

This study identified two candidate hub genes (*Sytl4* and *Kcnc2*), developed a nomogram for diagnosing the obesity‐associated risks in offspring and presented a dysregulated proportion of immune cells in the hypothalamus of offspring due to maternal obesity. This study provides potential candidate genes for determining obesity‐associated risks in offspring.

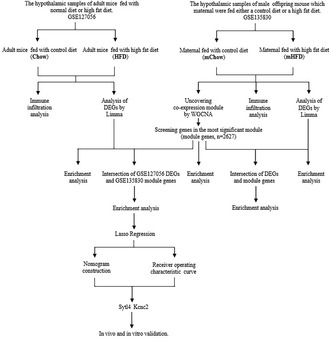

## 1 INTRODUCTION

Obesity is a global epidemic and a significant health concern among women of childbearing age in the 21st century. Increasing evidence shows that maternal obesity increases the risk of metabolic diseases such as obesity and type 2 diabetes mellitus (T2DM) in the offspring.^1^ Observational studies have indicated a strong association between maternal obesity and a higher risk of coronary heart disease, stroke, asthma and adverse neurodevelopmental outcomes.^2^ Multiple perinatal factors resulting from maternal obesity, including metabolic dysregulation, low‐grade inflammation, altered endocrine factors, placental dysfunction, and gut microbiome changes, can influence fetal growth and predispose offspring to various morbidities later in life.^2^ Therefore, it is critical to delve into the underlying mechanisms and predict the transmission of metabolic diseases to the offspring of obese mothers.

Metaflammation, a chronic low‐grade inflammatory state resulting from altered metabolism, is frequently found in obesity‐complicated pregnancies. It is increasingly acknowledged as an early‐life factor that influences offspring health.^3^ Mothers with pre‐pregnancy obesity tend to have high circulating levels of pro‐inflammatory markers/cytokines, such as IL‐8, IL‐6, CRP, TNF‐α, IFN‐γ, and altered adipokine level.^4^ Human data have revealed that the activated pro‐inflammatory state during pregnancy is linked to adverse health outcomes in offspring, including childhood obesity.^4^ Animal experiments have demonstrated that fetal inflammation induced by maternal obesity significantly contributes to the promoting adipogenesis and increasing adiposity in the offspring.^5^ Therefore, further investigation is needed to understand the immune‐associated mechanisms that lead to the metabolic disorders in offspring with maternal obesity.

Excessive energy intake, reduced energy consumption, abnormal energy storage, and destruction of energy homeostasis are the main causes of obesity.^6^ Energy homeostasis is a complex process governed by the hypothalamus, a central regulator of feeding behavior and energy expenditure. The arcuate (Arc) nucleus of the hypothalamus comprises anorexigenic neuropeptide Y (NPY)/agouti‐related protein (AgRP) neurons and anorexigenic proopiomelanocortin (POMC) neurons, which subtly regulate the energy balance.^7^ Recent evidence suggests that although neurons are fundamental to the physical functions of the hypothalamus, their proper function and systemic metabolic regulation largely rely on the surrounding non‐neuronal cells, such as glia, epithelial cells, pericytes, and endothelia.^8^ Aberrant nutritional elements, including fatty acids and glucose, transported into the hypothalamus primarily through the permeable blood–brain barrier (BBB), can induce glial activation and metaflammation within the hypothalamus.^9^ Hypothalamic inflammation is increasingly appreciated as an important mechanism for obesity and other metabolic disorders.^8^, ^9^

Perinatal obesogenic conditions can cause the abnormal neurodevelopment and neuroinflammation in the hypothalamus.^10^ Offsprings of high‐fat diet (HFD)‐fed mothers show elevated NPY expression and reduced POMC expression in the hypothalamus, leading to increased appetite and food intake.^1^ Increased intrauterine metabolic mediators, including glucose, insulin, leptin, and free fatty acid, could lead to multiple hypothalamic programing alterations, such as hypothalamic inflammation, defects of neuronal proliferation and endoplasmic reticulum stress (ERS).^1^ While mounting experimental evidence has underscored the central role of hypothalamic regulation in the metabolism of offspring exposed to maternal obesity, further investigations are necessary to elucidate the hub genes and underlying mechanisms.

The rapid advancement of high‐throughput sequencing technologies and bioinformatics has presented researchers with a valuable opportunity to unravel the regulatory mechanisms of disease progression. Weighted gene co‐expression network analysis (WGCNA), an unbiased systematic biological analysis, is extensively employed to delineate correlation patterns among genes across various samples. It evaluates the relationship between gene modules and relevant clinical features while identifying the core genes within the network.^11^, ^12^ The least absolute shrinkage and selection operator (LASSO) regression is a popular machine learning method known for its ability to effectively select important feature values with non‐zero coefficients through regularization. As a result, it is widely employed in the classification or feature selection of high‐dimensional data.^12^, ^13^ To our knowledge, a limited number of studies have combined WGCNA and LASSO regression to identify the key genes that reflect the effects of maternal obesity on the hypothalamic function of offspring.

In this study, we retrieved gene expression datasets of the hypothalamus from offspring with maternal obesity (GSE135830) and young adult mice fed a high‐fat diet (HFD) (GSE127056) from the Gene Expression Omnibus (GEO) database. Differentially expressed genes (DEGs) were analyzed by Limma. WGCNA was performed on GSE135830 to identify important module genes associated with offspring metabolic disorders in offspring affected by maternal obesity. Intersection analysis, enrichment analysis, Lasso algorithm, immune cell infiltration analysis, nomogram, and receiver operating characteristic (ROC) curve evaluation were subsequently performed to identify pivotal biomarkers for maternal obesity. Subsequently, immune infiltration analysis was conducted. Expression of the candidate genes was further validated in a mouse model of hypothalamic inflammation and a microglial inflammation model. This research could offer insights into the effects of maternal obesity on the offspring hypothalamus and potentially lead to the identification of potential immune‐associated markers for the obesity‐associated risk in the offspring with maternal obesity.

## 2 MATERIALS AND METHODS

### 2.1 Microarray data

**FIGURE 1:**
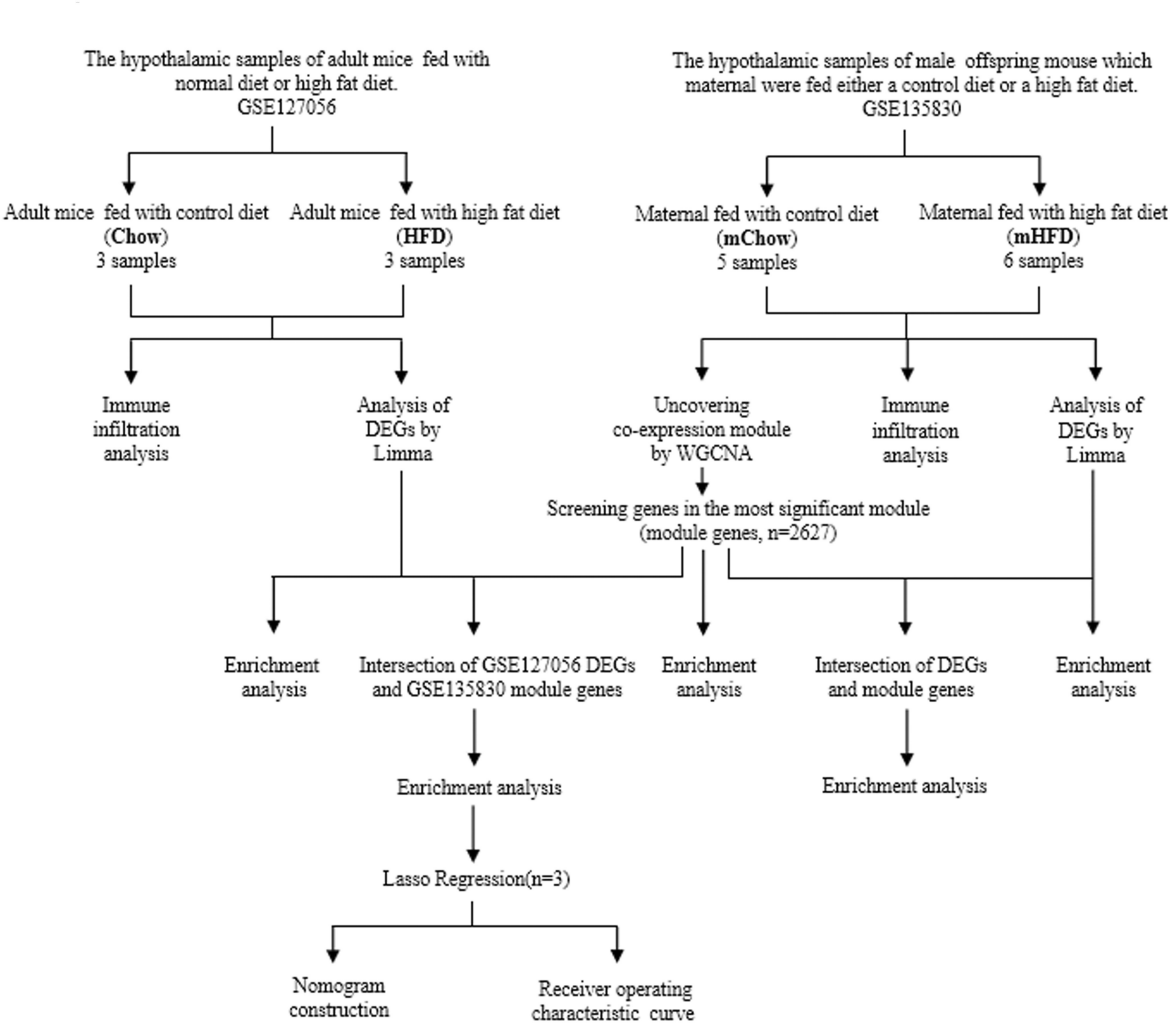
Study flowchart. DEGs, differentially expressed genes; GSE, gene expression omnibus series; Limma, linea models for microarray data; ROC, receiver operating characteristic; WGCNA, weighted gene coexpression network analysis.

Figure 1 shows a flowchart of the study. GSE135830, the gene expression data from the hypothalamus of the offspring of maternally obese mice fed a high‐fat diet (mHFD group) and a chow diet (mChow group) and GSE127056, including gene expression data from the hypothalamus of obese young adult mice fed an HFD and controls (HFD and Chow groups), were downloaded from the GEO (https://www.ncbi.nlm.nih.gov/geo/) database.

### 2.2 Data processing and differentially expressed gene (DEG) screening

Background calibration, normalization, and log2 transformation were performed on the GSE135830 and GSE127056 datasets using Limma in R. When multiple probes were used to identify the same gene, the average value was calculated to determine the expression level. According to previous studies on the transcriptomics of the diet‐induced obesity (DIO) animal models, the gene expression changes related to obesity are usually at a modest and even subtle level.^14^, ^15^ As previously described, a fold change (FC) above 1.5 (i.e., |log_2_FC| > 0.585) and *p* value < 0.05 were set as the criteria for identifying DEGs using the Limma package.^14^, ^15^

### 2.3 Weighted gene co‐expression network analysis (WGCNA)

To identify the modules related to maternal obesity, we conducted WGCNA^11^ on the offspring hypothalamus microarray database of the mChow and mHFD mouse dams from GSE135830. The median absolute deviation (MAD) of each gene was calculated and a free co‐expression network was constructed. Adjacency was computed by the co‐expression similarity‐derived “soft” thresholding power (β). This adjacency was then transformed into a topological overlap matrix (TOM) and the gene ratios and differences were analyzed. Modules were detected using hierarchical clustering and a dynamic tree‐cut function. We computed the dissimilarity of module eigengenes and selected a cut‐off line for the module dendrogram. Finally, the eigengene network was visualized. WGCNA was used to identify important modules in the offspring with maternal obesity.

### 2.4 Functional enrichment analysis

The gene ontology (GO) system offers structured, functional insights into genes,^16^ while the Kyoto Encyclopedia of Genes and Genomes (KEGG) serves as a comprehensive database housing signaling pathways related to gene functions.^17^ Functional enrichment analysis was performed utilizing the R package clusterProfiler,^18^ and the outcomes of the enrichment analysis were visualized using the ggplot2 R package. A significance criterion of *p* < 0.05 was established.

### 2.5 Regulatory interaction network analysis

We constructed a regulatory interaction network encompassing transcription factor‐target gene‐miRNAs to identify both transcriptional and post‐transcriptional regulatory factors of the genes under scrutiny. To build the regulatory interaction network, we utilized the web‐based Network Analyst tool.^19^

### 2.6 Machine learning

The LASSO is a regression method used for selecting variables to enhance the predictive accuracy and comprehensibility of a statistical model.^20^ We implemented Lasso regression analysis using the “glmnet” R package.^21^ Genes identified in the LASSO analysis were considered as candidate hub genes for diagnosis of obesity in offspring with maternal obesity.

### 2.7 Nomogram construction and receiver operating characteristic (ROC) evaluation

The “rms” R package was utilized to construct the nomogram from the candidate genes.^22^ “Points” means the score of candidate genes, while “Total Points” reflects the summation of all the scores of genes considered. Additionally, a receiver operating characteristic (ROC) curve was generated and calculated the area under the ROC curve (AUC) value was calculated to estimate the predictive utility of the identified biomarkers.

### 2.8 Immune infiltration analysis

Immune cell infiltration analysis was performed using the “Cibersort” R package.^23^ A bar plot was employed to visualize the proportion of each type of immune cell in various samples. The proportions of different types of immune cells in the maternal obesity and control groups were visualized using Vioplot. Furthermore, we generated a heatmap illustrating the correlation of 25 types of infiltrating immune cells was generated using the “corrplot” R package.^24^

### 2.9 High‐fat diet (HFD) induced mouse obesity model

Six‐week‐old male C57BL/6J mice were procured from the Experimental Animal Center of Nantong University. The animals were housed under diurnal lighting conditions with 12 h of light and had free access to food and water. The mice were randomly segregated into two groups (*n* = 6 per group) and given ad libitum access to water with either a high‐fat diet containing 60% kcal fat (HFD, D12492, Research Diets) or a normal chow diet (NCD, D12450J, Research Diets) for 4 weeks.^25^ Food intake and body weight were monitored weekly.

### 2.10 Glucose tolerance test

For the glucose tolerance test (GTT), mice were injected intraperitoneally (i.p.) with d‐glucose (1 g/kg, G7021, Sigma, USA) after 12 h of fasting. Blood glucose levels were determined in the tail vein at 0, 15, 30, 60, 90, and 120 min after injection.

### 2.11 Hypothalamus tissue collection

Following the GTT, the animals were anesthetized and euthanized by cervical dislocation to harvest the hypothalamus. The tissues were stored at −80°C until use. All animal experiments adhered to a protocol approved by the Institutional Animal Ethics Committee.

### 2.12 Cell culture and treatment

The mouse microglial cell line BV2 was purchased from Cellcook Biotech (Guangzhou, China) and cultured in Dulbecco's modified Eagle's medium DMEM‐low glucose (1.0 g/L) (C11885500BT, Gibco, USA) containing 10% fetal bovine serum (FBS) and 1% penicillin–streptomycin (Invitrogen, USA). To establish a metaflammation model in vitro, BV2 microglial cells were stimulated by palmitic acid (PA, 200 μmol/L) (Kunchuang Biotech, China) for 0, 3, 6, 12 or 24 h. Then, the cells were collected for further experiments.

### 2.13 Quantitative real‐time polymerase chain reaction (RT‐qPCR)

Total RNA from the mouse hypothalamic tissues and BV2 microglial cells was extracted using the TRIzol reagent (Invitrogen, USA) and quantified using a NanoDrop spectrophotometer (Thermo Fisher Scientific, USA). cDNA was synthesized using 5 × PrimeScript RT Master Mix (TaKaRa, Japan) following the manufacturer's instructions. RT‐qPCR was carried out using 2 × Low Rox SYBR Green (Beyotime, China) to evaluate gene expression using Bio–Rad CFX Maestro 1.0 system. The relative mRNA expression was analyzed by using the $2^{-∆∆C_{T}}$ method with *α‐tubulin* as the reference gene. The primer pair sequences are listed in Table 1.

**TABLE 1:** The primer pair sequences used in this study.

	Forward primer (5′–3′)	Reverse primer (5′–3′)
*α* *‐Tubulin*	GCATTAACTACCAGCCTCCCAC	CGCCTTCCACAGAATCCACAC
*Inos*	CAAGAGTTTGACCAGAGGACC	TGGAACCACTCGTACTTGGGA
*Il‐1* *β*	TCATTGTGGCTGTGGAGAAG	AGGCCACAGGTATTTTGTCG
*Sytl4*	TCCTGGGAGGGTTGAGTAGG	AGCAACCTGGGCAAGAAGAG
*Kcnc2*	GCCTCCTCCCACTCAACACT	CTGGTACCCCCGACATTGAG

### 2.14 Statistical analysis

ROC curves, AUC, and 95% CI values were obtained using SPSS Version 26.0 (IBM Corporation, Armonk, NY, USA). All body weight, GTT, and RT‐qPCR data were statistically analyzed using SPSS and visualized using GraphPad Prism 8.0.1 software. One‐way analysis of variance (ANOVA) was applied to compare differences between multiple groups. An unpaired *t‐*test was used to analyze the difference between the two groups and *p* < 0.05 indicating significant differences.

## 3 RESULTS

### 3.1 Identification and functional enrichment analysis of DEGs in adult mouse hypothalamus of HFD‐induced obesity model

**FIGURE 2:**
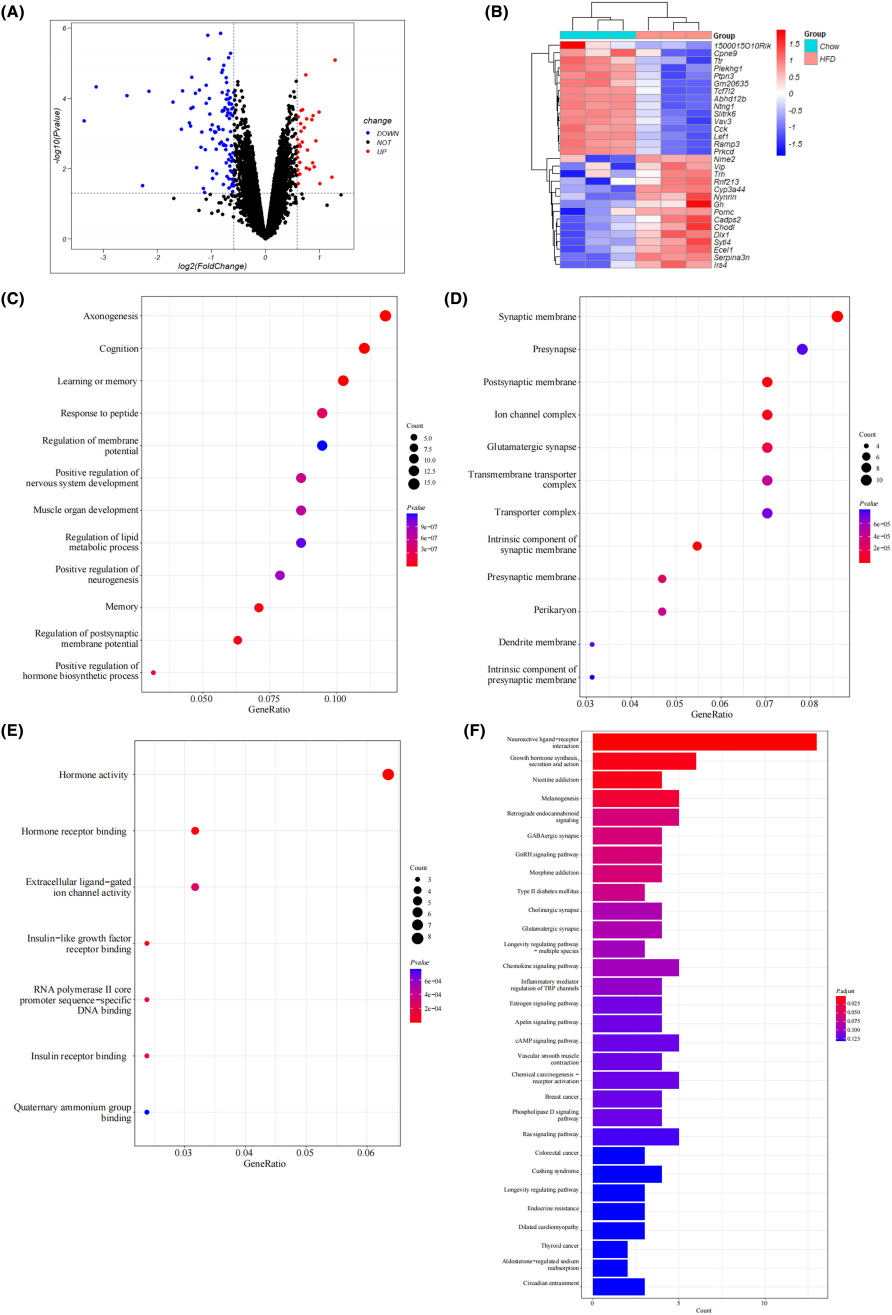
Identification and enrichment analysis of different expressed genes (DEGs) of the mouse hypothalamus between obesity and non‐obesity groups in GSE127056. (A) Volcano plot of DEGs. Red and blue dots represent DEGs with higher and lower expression levels in the mouse hypothalamus of the high‐fat diet (HFD) group compared with the Chow cases, respectively. (B) The heatmap shows the top 30 upregulated and downregulated DEGs, which are shown in red and blue colors, respectively. Each row shows the DEGs and each column refers to one of the samples of HFD cases or controls. (C–E) GO analysis of DEGs, including biological process (BP), cellular component (CC), and molecular function (MF). The *y*‐axis represents different GO terms, the *x*‐axis represents the gene ratio enriched in relative GO terms, the circle size refers to gene numbers, and the color represents the *p* value. (F) KEGG pathway analysis of DEGs. The *y*‐axis represents different signal pathway terms, the *x*‐axis represents the gene ratio enriched in relative KEGG terms, the bar length indicates gene numbers, and the color represents the *p* value.

To identify the differentially expressed obesity‐related genes in the mouse hypothalamus, we obtained GSE127056, an expression dataset from hypothalamic samples of young adult mice fed a high‐fat diet (HFD) or normal diet (chow). A total of 133 DEGs were identified in the hypothalamus of the HFD group compared to the control group; with 31 upregulated and 102 downregulated genes (Figure 2A,B). Functional enrichment analysis was performed to assess the functions of the DEGs. GO analysis revealed that DEGs were mainly enriched in biological process (BP) terms in the “axonogenesis” (Figure 2C) and in the “synaptic membrane” of the cellular component (CC) ontology (Figure 2D). The molecular function (MF) categories showed that “hormone activity” was the most important factor (Figure 2E). The KEGG analysis showed that DEGs were primarily enriched in the “neuroactive ligand‐receptor interaction” and “growth hormone synthesis, secretion, and action” (Figure 2F).

### 3.2 WGCNA and key module identification of maternal obesity offspring in GSE135830

**FIGURE 3:**
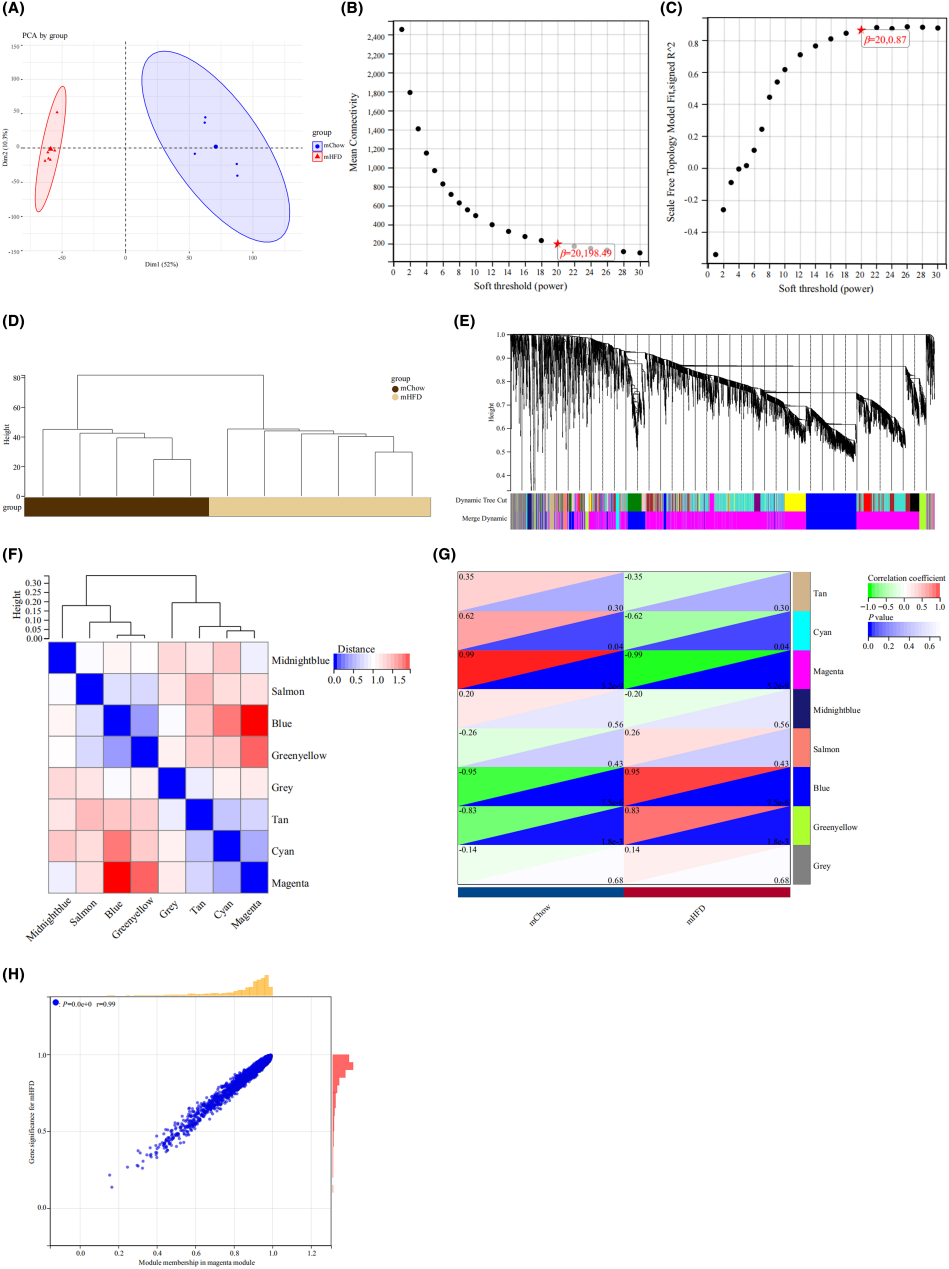
Identification of module genes related to maternal obesity offspring. WGCNA was performed on GSE135830, a microarray database of mouse hypothalamus from offsprings of maternal obesity. “mHFD” refers to the offspring of maternal obesity group in which the mouse dams fed high‐fat diet and “mChow” refers to the control offspring group of dams fed the chow diet. (A) Principal components analysis (PCA) score plot illustrates the distinction between mHFD and mChow groups. (B, C) *β* = 20 was chosen as the soft threshold based on the scale independence and average connectivity. (D) Clustering dendrogram displays the grouping of the mHFD and control samples. (E) Different colors represent gene co‐expression under the gene tree. (F) Heatmap of eigengene adjacency. (G) Heatmap of the association between modules and maternal obesity (mHFD). The magenta and blue modules demonstrate a significant correlation with mHFD. The numbers in the top and bottom brackets indicate the correlation coefficient and *p* value, respectively. (H) Correlation plot depicts the relationship between module membership and gene significance of magenta module genes.

To identify the modules most correlated to the hypothalamic features in offspring of maternal obesity, we performed WGCNA on GSE135830 (the microarray database of hypothalamic tissues from offspring of the mChow and mHFD mouse dams). The principal component analysis (PCA) was performed to determine the independence of the samples (Figure 3A). Hypothalamic samples from the offspring with maternal obesity (mHFD group) and control offspring (mChow group) exhibited obvious differences. We chose *β* = 20 (scale‐free *R* ^2^ = 0.87) as the “soft” threshold based on the scale independence and average connectivity (Figure 3B,C). Figure 3D depicts the clustering dendrogram between the mHFD offspring and mChow groups. Based on this power, eight gene co‐expression modules (GCMs) were established (Figure 3E,F). The correlation between mHFD offspring and GCMs is shown in Figure 3G and the magenta module (correlation coefficient = −0.99, *p* = 5.2*10^−9^) and blue module (correlation coefficient = 0.95, *p* = 7.5*10^−6^) contain 2627 genes that demonstrated the highest correlation with mHFD offspring. These genes were regarded as the pivotal modules for subsequent analyses.

Furthermore, we computed the correlations between module membership and gene significance in the magenta and blue modules for the mHFD offspring traits. As expected, a significant positive correlation was observed among the magenta modules (*r* = 0.99; Figure 3H). The correlation ratio of the blue module between the mHFD offspring and mChow control groups was 0.95 (data not shown). Therefore, we determined that these 2627 genes in magenta and blue modules are most significantly associated with the characteristics of the offspring hypothalamus with maternal obesity.

### 3.3 Functional enrichment analysis of WGCNA module genes related to maternal obesity offspring

**FIGURE 4:**
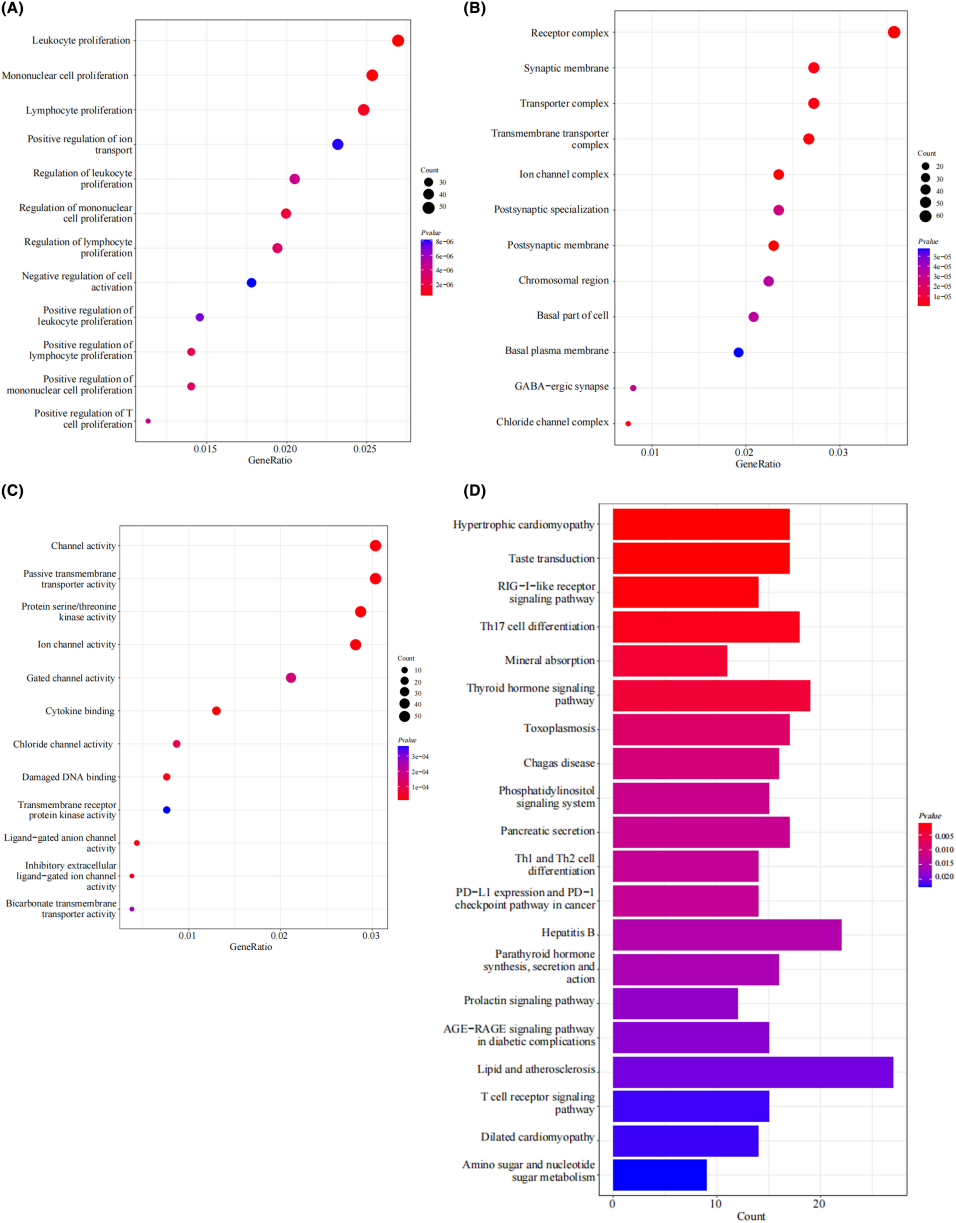
Functional enrichment analysis of WGCNA module genes related to maternal obesity offspring. (A–C) GO analysis of the 2627 genes in the magenta and blue modules selected by WGCNA on GSE135830, including biological process (BP), cellular component (CC) and molecular function (MF). (D) KEGG pathway analysis of the magenta and blue module genes.

Subsequently, we conducted a functional enrichment analysis to annotate the potential functions of the 2627 genes in the magenta and blue modules identified by WGCNA. The GO analysis revealed that these maternal obesity‐related module genes were mainly enriched in BP terms, including “leukocyte proliferation,” “mononuclear cell proliferation,” and “lymphocyte proliferation” (Figure 4A). Regarding the CC ontology, the maternal obesity‐related module genes were mainly located in the “receptor complex,” “synaptic membrane,” and “transporter complex” (Figure 4B). The MF analysis showed that “channel activity,” “passive transmembrane transporter activity,” and “protein serine/threonine kinase activity” were the most significant items among the module genes (Figure 4C). The KEGG analysis showed that the maternal obesity correlated module genes were primarily enriched in “lipid and atherosclerosis,” “hepatitis B,” and “thyroid hormone signaling pathway” (Figure 4D).

### 3.4 Common genes of WGCNA modules and DEGs in offspring hypothalamus of maternal obesity in GSE135830

**FIGURE 5:**
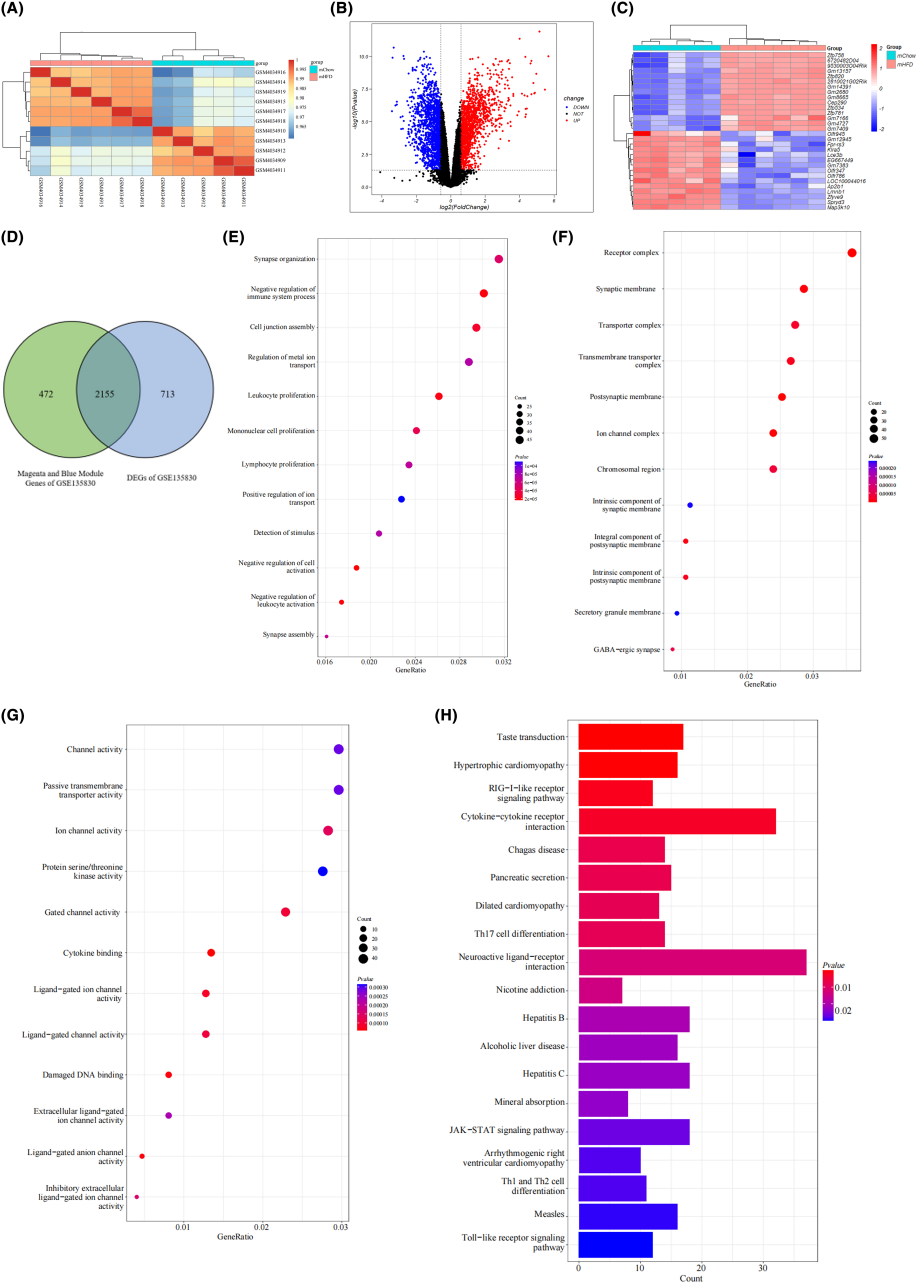
Functional enrichment analysis of the intersection genes of WGCNA key modules and DEGs of maternal obesity in GSE135830. (A) Heatmap of samples. Cyan represents the hypothalamic samples of the mChow offspring and red represents the mHFD offspring. (B) Volcano plot of DEGs. Red and blue dots represent DEGs with upregulated and downregulated expression level in the hypothalamus of the mHFD versus mChow group, respectively. (C) Heatmap of the top 30 DEGs. Each row shows the DEGs and each column refers to one of the samples of mHFD offsprings or mChow controls. (D) Venn diagram of 2155 common genes which were identified from the intersection of DEGs and WGCNA key module genes of maternal obesity. (E–G) GO analysis of the common genes. (H) KEGG pathway analysis of the common genes.

To investigate the impact of maternal obesity on the offspring hypothalamus, we screened the DEGs between the offspring hypothalamus from the mHFD and mChow groups in GSE135830. A total of 2868 DEGs (1412 upregulated and 1456 downregulated genes) were identified between the mHFD offspring and the mChow samples. Figure 5A displays a heatmap of the samples, illustrating a clear distinction between the HFD and control groups. Figure 5B,C depict a heatmap and volcano plot, respectively, of GSE135830 DEGs.

To identify the DEGs mostly associated with maternal obesity, we determined the common genes between the key WGCNA modules (magenta and blue) and the DEGs of mHFD offspring in GSE135830. A total of 2155 common genes were identified by determining the intersection of 2868 DEGs and 2627 genes in the magenta and blue modules (Figure 5D). We then performed a functional enrichment analysis to determine the functions of these maternal obesity‐related 2155 DEGs in the hypothalamus of the offspring. The GO analysis revealed that the maternal obesity‐related DEGs were enriched in BP terms in the “synapse organization” and “negative regulation of immune system process” categories (Figure 5E). In the CC ontology, genes were mainly located in the “receptor complex” and “synaptic membrane” (Figure 5F). The MF analysis showed that “channel activity” was the most important item (Figure 5G). The KEGG analysis showed that DEGs were primarily enriched in the “neuroactive ligand–receptor interaction” and “cytokine–cytokine receptor” (Figure 5H).

### 3.5 Common genes of young adult obesity (GSE127056) and maternal obesity offspring (GSE135830)

**FIGURE 6:**
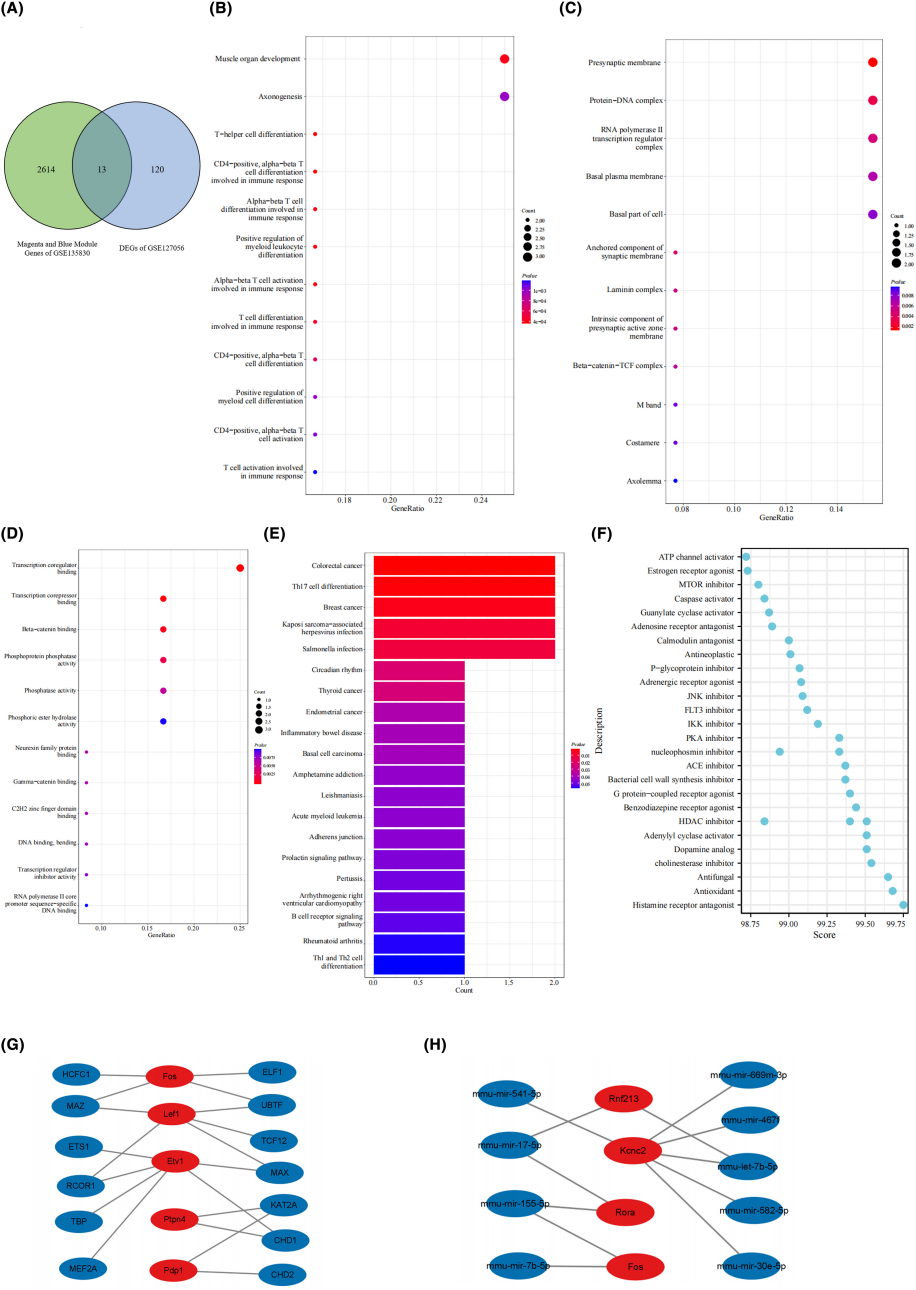
Functional enrichment analysis of the common genes of the WGCNA module linked to maternal obesity offspring in GSE135830 and DEGs of adult obesity in GSE127056. (A) A Venn diagram demonstrates the identification of 13 common genes resulting from the intersection of DEGs from GSE127065 and WGCNA module genes from GSE135830. (B–D) GO analysis of the intersection genes, including biological process, cellular component and molecular function. (E) KEGG pathway analysis of the intersection genes. (F) The dot plot shows the predicted compounds that may act on the 13 common genes by clue.io. The *y*‐axis represents different compounds and the *x*‐axis represents the score of compounds. (G, H) Gene regulatory network with transcription factor–gene interactions and gene–miRNA interactions constructed using the Network Analyst online tool. The red dot represents the 13 common genes and the blue represents the predicted transcription factors and miRNAs.

To assess the relationship between maternal obesity and offspring obesity, we conducted an intersection analysis to identify the common genes between the DEGs in the hypothalamus of the young adult obese mice (GSE127056) and the WGCNA module genes closely associated with the offspring hypothalamic traits in maternal obesity (GSE135830). A total of 13 common genes were screened via the intersection of 133 DEGs in GSE127056 and 2627 key module genes in the magenta and blue modules of GSE135830 (Figure 6A). Functional enrichment analysis was performed to clarify the roles of the 13 common genes. The GO analysis revealed that they were mainly enriched in the BP terms “muscle organ development” and “axonogenesis” (Figure 6B). Regarding the CC ontology, the 13 genes were mainly located in the “presynaptic membrane” and “protein–DNA complex” (Figure 6C). MF analysis showed that “transcription coregulator binding” and “transcription corepressor binding” were the most significant factors (Figure 6D). The KEGG analysis showed that they were primarily enriched in “Colorectal cancer” and “Th17‐cell differentiation” (Figure 6E). Moreover, we predicted the compounds potentially acting on the 13 genes using Clue.io online tools (Figure 6F). Furthermore, we constructed gene regulatory networks depicting transcription factor–gene interactions and miRNA–gene interactions using the Network Analyst online tool (Figure 6G,H).

### 3.6 Identification of candidate hub genes for the maternal obesity‐induced offspring metabolic disorder via machine learning and diagnostic value assessment

**FIGURE 7:**
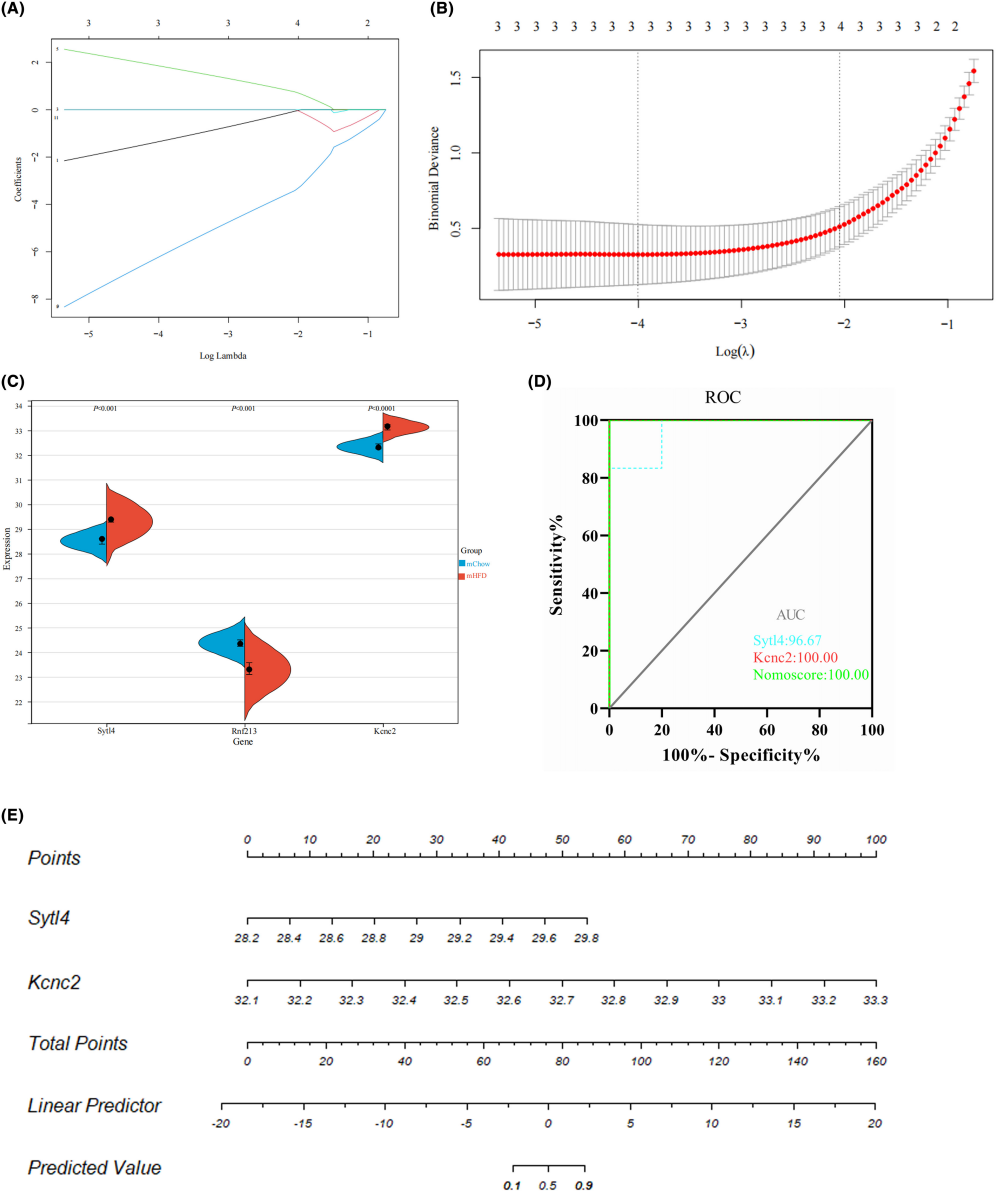
Machine learning screening of candidate biomarkers for offspring obesity induced by maternal obesity. (A, B) Biomarker screening in the Lasso model. The number of genes (*n* = 3) corresponding to the lowest point of the curve is the most suitable for offspring obesity diagnosis. (C) Split violin plots of the three candidate biomarkers. Red represents the mHFD offspring and blue represents the mChow offspring. (D, E) The ROC curve of each candidate gene (*Sytl4* and *Kcnc2*) and nomogram show a significant diagnostic value for offspring obesity.

To identify the hub genes associated with the risk of obesity development in the offspring of mothers with obesity, we applied LASSO regression machine learning algorithms to the 13 common genes. This approach allowed us to screen candidate genes for nomogram construction and evaluating diagnostic values. As shown in Figure 7A,B, the LASSO regression algorithm identified three potential candidate biomarkers (Synaptotagmin‐like 4 (*Sytl4*), Ring finger protein 213 (*Rnf213*), and potassium voltage‐gated channel Shaw‐related subfamily member 2 (*Kcnc2*)) for offspring with maternal obesity. We assessed the expression of these three genes in the GSE135830 dataset using a split‐violin plot (Figure 7C). The *Sytl4* and *Kcnc2* genes were upregulated and *Rnf213* was downregulated in the mHFD group. Receiver operating characteristic (ROC) curves were established to assess diagnostic specificity and sensitivity. We calculated the AUC and 95% CI for each gene: *Sytl4* (AUC 0.967, CI 0.868–1.000), *Kcnc2* (AUC 1.000, CI 1.000–1.000), *Rnf213* (AUC nonsignificant, data not shown) (Figure 7D). Therefore, we constructed a nomogram based on the two candidate hub genes (*Sytl4* and *Kcnc2*) and assessed its diagnostic value with ROC (AUC 1.000, CI 1.000–1.000) (Figure 7D,E). These results indicated that each candidate gene may possess a high biomarker value for offspring obesity induced by maternal obesity and that the constructed nomogram had the highest diagnostic value.

### 3.7 Immune cell infiltration analysis

Increasing evidence suggests that hypothalamic inflammation plays a pivotal role in disrupting the homeostatic control of energy balance, thereby increasing the vulnerability to metabolic disorders in the offspring of obese mothers.^8^ In line with this, functional enrichment analysis revealed that the offspring obesity‐associated genes were not only enriched in the physical functions of the hypothalamus (such as neurodevelopment and metabolic hormone regulation) but also in immune regulation (such as immune cell differentiation and cytokine‐cytokine receptor activation) (Figure 4, 5, 6). Thus, immune cell infiltration analysis was performed to better clarify the immune regulation in the hypothalamus of offspring with maternal obesity.

**FIGURE 8:**
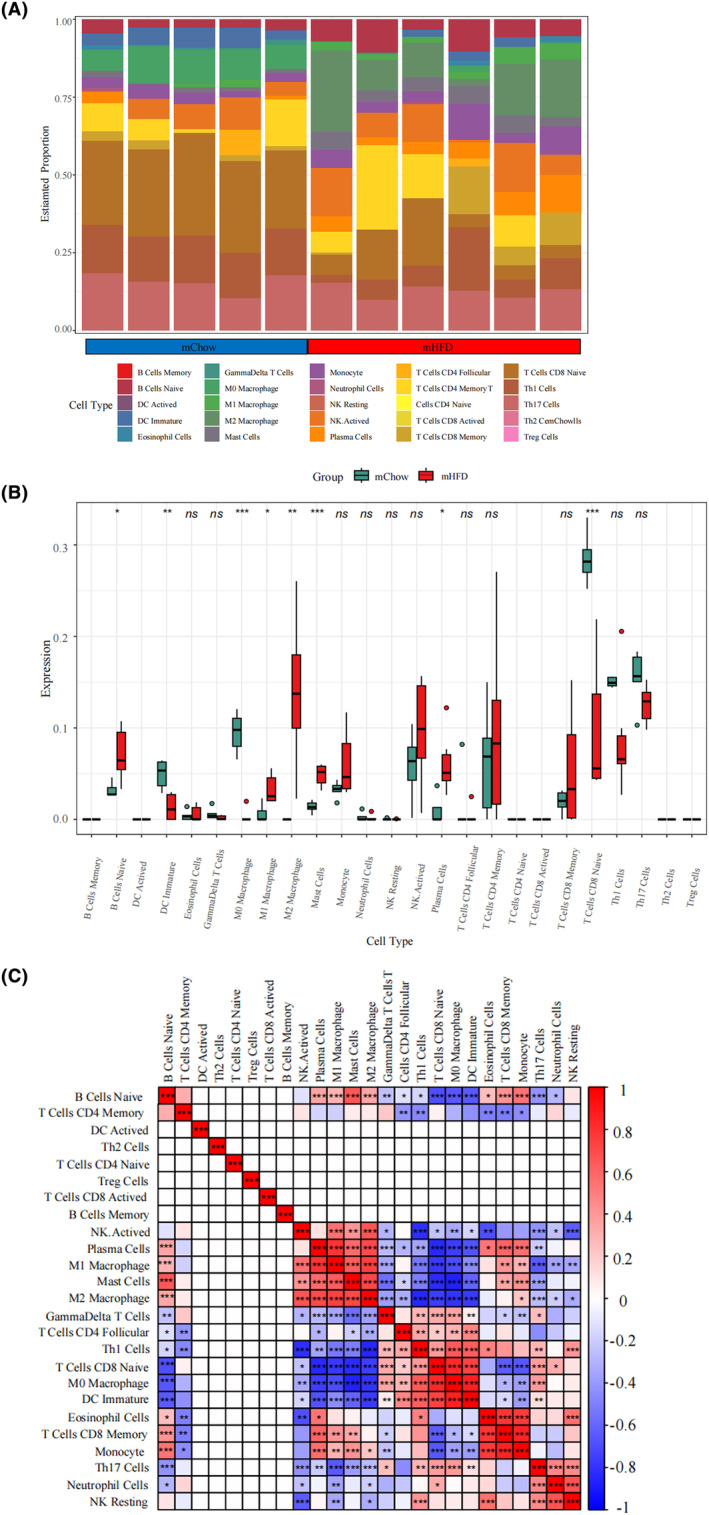
Immune cell infiltration analysis between the mHFD offspring and mChow control groups. (A) Barblot showing the proportion of 25 immune cell types in different samples. (B) Boxplot showing the comparison regarding the proportions of 25 immune cell types between the mHFD offspring and mChow control groups. (C) Correlation of the compositions of 25 immune cell types. *p < 0.05, **p < 0.01, ***p < 0.001.

The barplot (Figure 8A) displays the proportion of the 25 immune cell types in each sample for both the mHFD and mChow offspring in GSE135830. The mHFD offspring exhibited an elevated level of naive B cells, plasma cells, mast cells, as well as M1 and M2 macrophages and reduced levels of naive CD8 T cells, immature DC and M0 macrophages in the hypothalamus compared to the mChow group (Figure 8B). The correlation of 25 types of immune cells revealed that T cells CD8 Memory were positively associated with monocytes (*r* = 0.85) and T cells CD8 Naive were positively correlated with M0 macrophages (*r* = 0.84), whereas M0 macrophages were negatively related to mast cells (*r* = −0.87) (Figure 8C). Regarding the HFD‐induced adult obesity model (GSE127056), no significant difference in immune cell infiltration was detected between the hypothalamus of HFD mice and the control group (Figure S1). In conclusion, a variety of immune cell types exhibited distinct infiltration patterns in the hypothalamus during obesity. These findings suggest a potential contribution to hypothalamic inflammation in offspring with maternal obesity, highlighting a potential regulatory target for addressing early obesity risk.

### 3.8 In vivo and in vitro validation of hub genes

**FIGURE 9:**
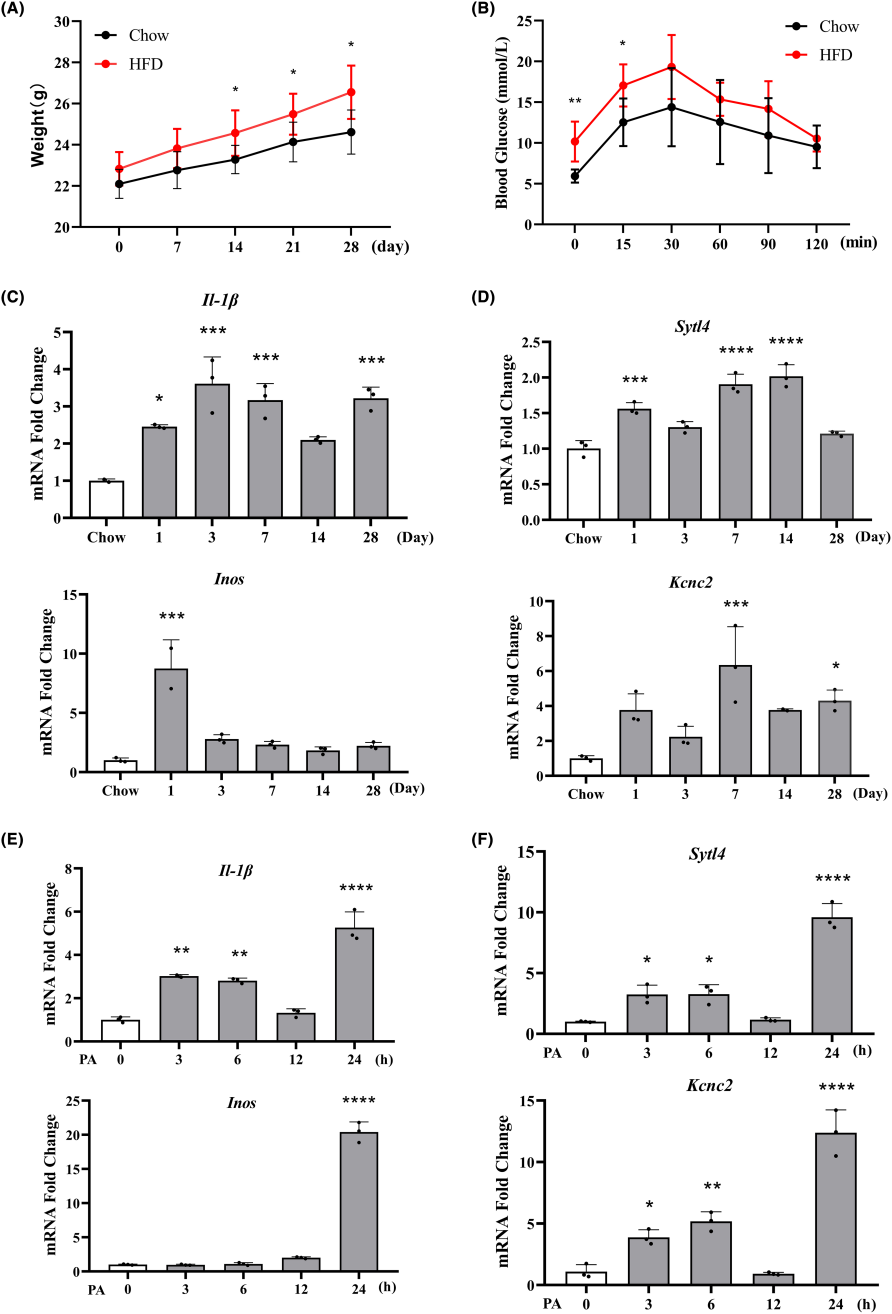
Validation of the hub gene expression in hypothalamic inflammation of obesity in vivo and in vitro. (A, B) The mice were fed with either a normal chow diet (Chow) or a high‐fat diet (HFD) for 28 days. Changes in body weight and glucose tolerance tests after 12‐h fasting. (C, D) The mRNA expression of proinflammatory markers (*Il‐1β* and *Inos*) and two hub genes (*Sytl4* and *Kcnc2*) was analyzed by RT‐qPCR in the mouse hypothalamus, respectively. (E, F) Mouse microglial cell BV2 was treated with palmitic acid (PA, 200 μmol/L) and the mRNA expression of *Il‐1β*, *Inos* and the screened hub genes (*Sytl4* and *Kcnc2*) was assessed using RT‐qPCR. Compared with the control group: *p < 0.05, **p < 0.01, ***p < 0.001, ****p < 0.0001.

To confirm the expression of the selected hub genes during the hypothalamic inflammation in obesity, we established a high‐fat diet (HFD)‐induced hypothalamic inflammation mouse model. The body weight on the 28th day compared to the control group fed a normal chow diet (Chow), indicating an early phase of HFD‐induced obesity (Figure 9A). Accordingly, the blood glucose levels of both fasting and 15 min GTTs were significantly increased in the HFD group compared to those in the chow‐fed group (Figure 9B), suggesting weakened glucose tolerance. RT‐qPCR assays proved that the mRNA expression of the pro‐inflammatory markers *Il‐1β* and *Inos* was significantly elevated in the mouse hypothalamus following HFD administration, indicating the successful establishment of an HFD‐induced hypothalamic inflammation model (Figure 9C). Furthermore, RT‐qPCR confirmed the increased mRNA expression of both *Sytl4* and *Kcnc2* in the hypothalamus of the HFD group, consistent with the microarray analysis results (Figure 9D).

As tissue‐resident macrophages in the brain, microglia play a pivotal role in hypothalamic inflammation.^26^ The microglial metaflammation model was induced by stimulating BV2 microglial cells with palmitic acid (PA, 200 μmol/L) in vitro. RT‐qPCR assays revealed a significant increase in the mRNA expression of *Il‐1β* and *Inos* in response to PA, indicating the inflammatory activation of microglia (Figure 9E). Meanwhile, *Sytl4* and *Kcnc2* expression were also elevated in BV2 cells following PA administration, which further verified the bioinformatic analysis results (Figure 9F). These results indicate that *Sytl4* and *Kcnc2* may act as hub genes that participate in overnutrition‐induced microglial activation and hypothalamic inflammation during obesity.

## 4 DISCUSSION

Maternal obesity is a major concern for mothers and their offspring. In addition to the strong correlation with negative health outcomes during pregnancy and birth, such as stillbirth and preeclampsia, maternal obesity could also lead to long‐term consequences for offspring, predisposing or “programing” them to the development of metabolic disease in adulthood.^5^ Changes in the development of the hypothalamus, a regulatory center for energy homeostasis and metabolic balance, are increasingly recognized as critical determinants of the offspring risk of metabolic disorders; however, the underlying mechanisms are only beginning to be understood.^7^ Here, through integrated bioinformatics analyses and machine learning methods, we identified two pivotal immune‐associated candidate genes (*Sytl4* and *Kcnc2*) and constructed a nomogram for diagnosing the obesity‐associated risk in the offspring of mothers with obesity.

### 4.1 Maternal obesity impairs the hypothalamic development of the offspring

Maternal obesity reportedly affects several key steps in hypothalamic neurodevelopment in the offspring. First, maternal obesity may disturb neuronal proliferation and differentiation in the hypothalamus of the offspring. Offspring from obese mothers show diminished proliferation of neural progenitor cells (NPCs), as indicated by the decreased levels of the proliferation marker Ki67.^27^ Exposure to maternal obesity activates Notch signaling and represses proneural factors, such as Mash1 and Ngn2/3, which are critical for neurogenesis in the hypothalamus of offspring.^28^ Second, maternal obesity may influence the formation of functional neuronal networks in the hypothalamus.^1^ Offspring of mice with maternal obesity show altered expression of markers for neurogenesis, axogenesis and synaptic plasticity in the hypothalamus, which might explain the pathogenesis of hypothalamic feeding network dysfunction in the offspring due to maternal obesity.^29^ Third, maternal obesity may weaken nutrient sensing in the hypothalamus of offspring, which is essential for the correct regulation of energy homeostasis. For example, exposure to maternal obesity causes hypothalamic insulin resistance in offspring during both during the in‐utero and post‐natal period.^30^

In this study, GO analysis revealed that the maternal obesity‐related DEGs in offspring hypothalamus are mainly enriched in “axonogenesis,” “synaptic membrane” and “channel activity”; KEGG analysis discovered that they were primarily associated with the “neuroactive ligand–receptor interaction.” Therefore, our findings suggest that exposure to maternal obesity significantly influences the hypothalamic development of offspring. This impact may occur through the reduction of NPC proliferation, impairment of hypothalamic projection formation, and alteration of neurotrophic factor signaling.

### 4.2 Maternal obesity causes the hypothalamic inflammation in offspring

Recent evidence suggests that the proper development and function of hypothalamic neurons, such as orexigenic NPY/AgRP and anorexigenic POMC neurons within the ARC, are subtly influenced by the surrounding non‐neuronal cells.^8^, ^31^ The perinatal obesogenic milieu of maternal obesity leads to hypothalamic inflammation, which is a crucial mechanism for metabolic dysregulation in offspring.^10^ Alleviating hypothalamic inflammation with cannabidiol significantly enhanced the peripheral metabolic profile of Wistar rat offspring.^32^ A prospective human dual‐cohort study observed an approximately linear association between maternal pre‐pregnancy BMI and offspring hypothalamic microstructure on MRI images, further demonstrating the plasticity of prenatal hypothalamic development in the context of maternal overnutrition during pregnancy.^33^

Interestingly, in the present study, the hypothalamic DEGs of offspring with maternal obesity were not only enriched in the physical functions of the hypothalamus (such as neurodevelopment and metabolic hormone regulation) but also in immune system regulation. The hypothalamic DEGs related to maternal obesity were primarily enriched in “negative regulation of immune system process,” “receptor complex,” and “cytokine–cytokine receptor.” The shared hypothalamic DEGs of offspring with maternal and adult obesity were also closely involved in immune cell differentiation and signal transduction. Therefore, our data indicate that hypothalamic inflammation may serve as a shared immunological mechanism for both overnutrition‐induced adult obesity and the metabolic disorders in offspring resulting from maternal obesity.

### 4.3 Immune cell infiltration in offspring hypothalamus caused by maternal obesity

Microglia, which are tissue‐resident macrophages in the brain, play an important role in the hypothalamic inflammation associated with obesity. The offspring of obese mothers, expressed elevated expression of ionized calcium‐binding adaptor molecule 1 (Iba1), a marker for microglia activation, in the hypothalamus.^1^ In a maternal high‐fat diet (mHFD) mouse model, elevated pro‐inflammatory IL‐6 stimulated the astrogliosis in the fetal and early neonatal hypothalamus.^34^ Recent studies of post‐mortem human tissue revealed that in addition to glia within the CNS, peripheral immune cells, including macrophages and even circulating T/B lymphocytes, can also infiltrate the cerebral parenchyma and participate in hypothalamic inflammation in human obesity.^35^, ^36^ The peripheral immune cell infiltration into the CNS may result from the leaky blood–brain barrier (BBB) at the median eminence–arcuate nucleus (ME–ARC) interface following the high‐energy diet consumption. A mouse model of bone marrow chimerism showed increased peripheral immune cell entry into the CNS induced by HFD consumption.^37^ In a mouse model of maternal obesity, HFD damaged the structure and function of the ME blood–brain barrier in the progeny.^38^

In this study, we observed that compared to mChow offspring, the hypothalamus of mHFD offsprings exhibited a higher level of naive B cells, plasma cells, mast cells, M1 and M2 macrophages, along with lower levels of CD8 naive T cells, immature DC, and M0 macrophages. This suggests infiltration and activation of both the innate immune cells (macrophages, DC, and mast cells) and adaptive immune cells (such as plasma cells).

It needs to be clarified that the “macrophage” population in the present assay may contain both the resident microglia within the CNS and peripheral macrophages infiltrated into the hypothalamus. Similar to peripheral macrophages, microglia also undergo M1/M2 phenotype polarization following different immunostimulants and participate in multiple CNS disorders, such as neurodegeneration, stroke, and traumatic brain injuries.^39^, ^40^, ^41^ Microglial polarization has also been suggested as a therapeutic target for manipulating hypothalamic inflammation and metabolic diseases. For example, electroacupuncture for weight loss reportedly regulates microglial polarization in the arcuate nucleus of the hypothalamus.^42^ In this study, the hypothalamus of mHFD offspring exhibited elevated levels of both M1 and M2 macrophages, whereas no significant difference was observed in the adult obesity mouse model. Different immune infiltration profiles may imply different immunostimulants. Compared to HFD‐induced obesity in adults, the perinatal obesogenic environment associated with maternal obesity may trigger hypothalamic inflammation in offspring through a more complex interplay of metabolic, hormonal, and immunological factors.

### 4.4 Hub genes for metabolic disorders in offspring due to maternal obesity

Using integrated bioinformatics analysis and machine learning methods, we identified two hub genes (*Sytl4* and *Kcnc2*) and developed a nomogram to assess obesity risk in offspring exposure to maternal obesity.

Synaptotagmin‐like 4 (*Sytl4*), also known as Synaptotagmin‐like Protein 4 (*Slp4*) or granuphilin, encodes a member of the synaptotagmin‐like protein family. This protein is characterized by an N‐terminal Rab27‐binding domain and a C‐terminal tandem C2 domain. Sytl4 interacts with specific small Rab GTPases and participating in intracellular membrane trafficking and regulating vesicle exocytosis and exosome secretion.^43^ Sytl4, as a GTP‐specific effectors of Rab27a, regulates insulin secretion in pancreatic β‐cells.^44^ MiRNA‐9 controls insulin secretion by targeting Granuphilin/Sytl4 expression.^45^ Sytl4 also regulates the age‐dependent exocytosis of secretory vesicles in PC12 neuroendocrine cells,^46^ and governs platelet secretion by α‐granule and dense granule secretion.^47^ Interestingly, *Sytl4* has been identified as a sexually dimorphic hub gene in the mouse hypothalamus,^48^ indicating its potential involvement in the physical hypothalamic function.

To our knowledge, the expression and function of Sytl4 during hypothalamic inflammation and obesity have not been investigated before. In this study, we identified *Sytl4* as a hub gene for metabolic disorders in the offspring of obese mothers. The in vivo HFD‐induced obesity model and the in vitro PA‐induced microglial inflammation model both verified the increased expression of *Sytl4*, indicating its potential role in hypothalamic inflammation in obese offspring.

The potassium voltage‐gated channel Shaw‐related subfamily member 2 (Kcnc2) also known as Voltage‐Gated Potassium Channel Kv3.2 (KV3.2), is a member of the voltage‐gated potassium channel subfamily. Kcnc2 is essential for generating of fast‐spiking properties in cortical GABAergic interneurons. Multiple Kcnc2 variations have been associated with epileptic encephalopathy.^49^ DNA microarray analysis performed on a spontaneously hypertensive rat model identified *Kcnc2* as a key gene linked to the onset of hypertension.^50^ Kcnc2 encodes the KV3.2 channels, primarily responsible for generating of the fast delayed rectifier (FDR) potassium current in suprachiasmatic nucleus (SCN) neurons, thereby regulating the intrinsic circadian rhythms generation.^51^ More interestingly, an integrative study identifies KCNC2 as a novel predisposing factor for childhood obesity and the risk of diabetes in the Korean population; Kcnc2 is associated with modified hepatic gluconeogenesis and increased ER stress in obesity‐mediated diabetic risk.^52^ A recent study identified Kcnc2 as a thermogenic gene in adipocytes. The PKA‐p38 MAPK‐KCNC2‐UCP1 signaling pathway is a potential target for obesity treatment.^53^

The expression and potential function of Kcnc2 in hypothalamus programing and inflammation in obesity remained unknown. Here, we identified *Kcnc2* as a hub gene for determining obesity risk in offspring with maternal obesity. Consistent with the findings of the bioinformatics analysis elevated expression of *Kcnc2* was confirmed in both the HFD‐induced obesity model and the in vitro microglial metainflammation model, suggesting its potential involvement in overnutrition‐induced immunologic dysregulation in the offspring hypothalamus of offspring. Further experiments are needed to explore its biological function as an immune‐associated hub gene linked to the risk of maternal obesity in offspring.

### 4.5 Limitation

Our study had several limitations. First, the number of hypothalamic samples remained low, and the diagnostic value of the nomogram was high because of the limited sample size. These results need to be confirmed in a larger‐scale study with a large sample size. Secondly, although we screened two candidate hub genes, their specific functions and underlying mechanisms have not yet been elucidated. It is essential to clarify their detailed contributions to hypothalamic programing and immunological mechanisms in obese offspring in future studies.

## 5 CONCLUSION

Our study systematically identified two candidate hub genes (*Sytl4* and *Kcnc2*) and developed a nomogram for diagnosing the obesity‐associated risks in offspring through various bioinformatics analyses and machine learning algorithms. We also noted a dysregulated proportion of immune cells in the hypothalamus of offspring due to maternal obesity. Our study provides potential diagnostic candidate genes for determining obesity‐associated risks in offspring.

## AUTHOR CONTRIBUTIONS

DZ and HL contributed to the hypothesis development and manuscript preparation. YS and XW contributed to the study design, investigation, data analysis and image processing. Others contributed to the data analysis. All the authors drafted the manuscript and approved its submission for publication.

## FUNDING INFORMATION

This work was supported by the Natural Science Foundation of Jiangsu Province (BK20211108, BK20221274); Scientific Research Project of Health Commission of Jiangsu Province (M2021106); Nantong Science and Technology Project (JC2021015); Jiangsu Provincial Medical Key Discipline (Laboratory) Cultivation Unit (JSDW202249); Scientific Research Innovation Team of Kangda College of Nanjing Medical University (KD2022KYCXTD005); Nantong Municipal Medical Key Laboratory of Molecular Immunology; Nantong Municipal Key Laboratory of Metabolic Immunology and Disease Microenvironment; Scientific Research Project of Health Commission of Nantong (MS2022025, MSZ2022016).

## CONFLICT OF INTEREST STATEMENT

The authors declare that they have no conflict of interest.

## Supporting information


Figure S1.

## Data Availability

The data that support the findings of this study are openly available in GEO DATABASE at https://www.ncbi.nlm.nih.gov/geo/, reference number GSE135830 and GSE127056.
