# Supplementary material for: Identifying of immune‐associated genes for assessing the obesity‐associated risk to the offspring in maternal obesity: A bioinformatics and machine learning
Source: CNS Neurosci Ther. 2024 Mar 27;30(3):e14700. doi: 10.1111/cns.14700 (PMC10973700; doi:10.1111/cns.14700)
Supplement: Supplementary file 1 — Figure S1. [file CNS-30-e14700-s001.zip › FigureS1.docx]

**Supplementary Materials**

**FigureS1 Immune Cell Infiltration Analysis of GSE127056.**

For the HFD and control groups, the proportions of 25 types of immune cells in each sample are shown in the barplot (**Figure S1A**). No significant differences were observed between the HFD and control groups (**Figures S1B)**. The correlation of 25 types of immune cells revealed that resting NK cells were positively associated with monocytes (*r*=0.81), whereas Treg cells were negatively associated with M1 macrophages (*r*= -0.98) **(Figure S1C).**


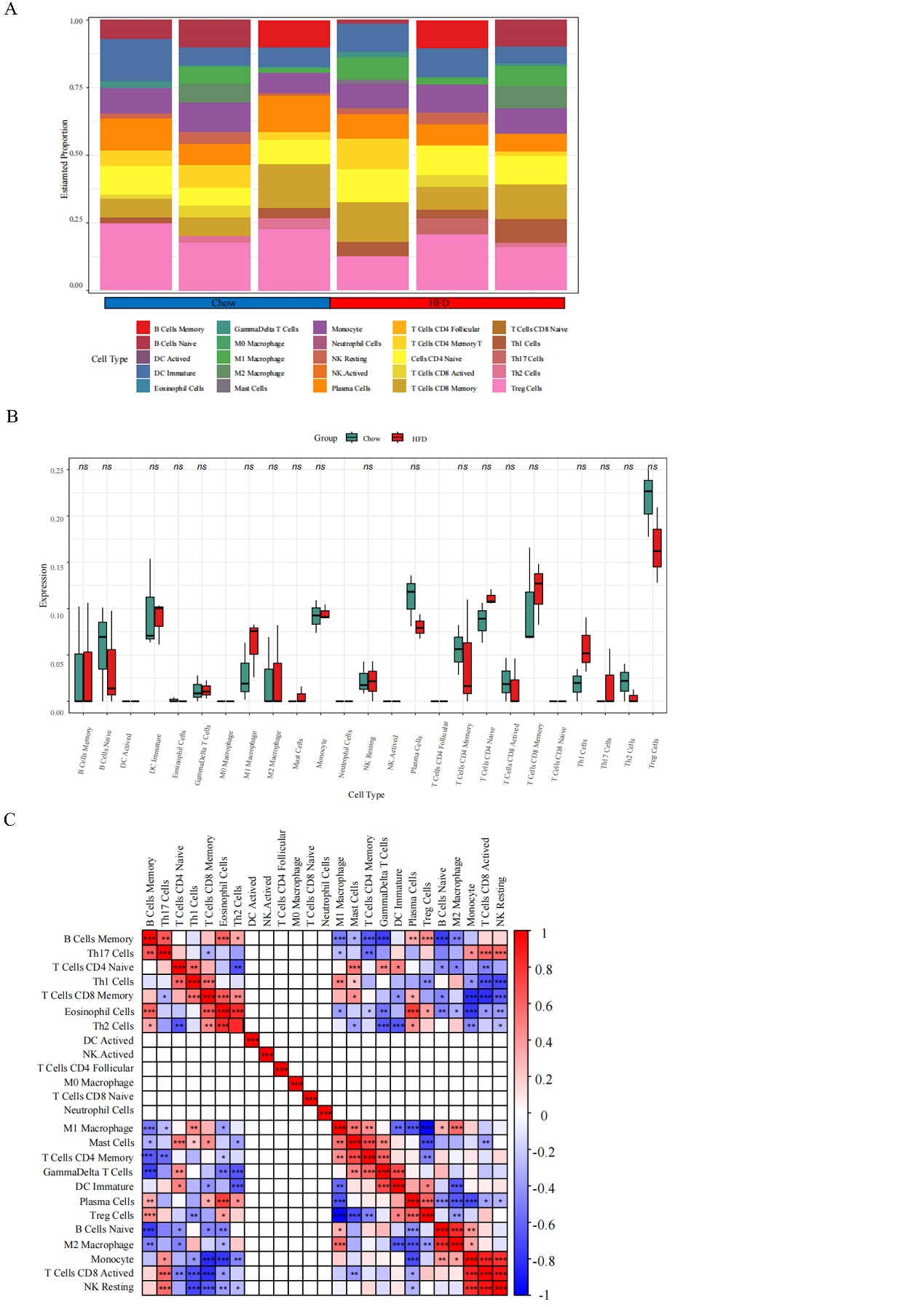
**Figure S1** | Immune cell infiltration analysis between the HFD and control groups of GSE127056. **(A)** The composition of 25 types of immune cells in every sample is visualized using the barblot. **(B)** The comparison regarding the proportion of 25 kinds of immune cells between the HFD and control groups visualized by the boxplot. **(C)** Correlation of 25 immune cell type compositions. **P* < 0.05, ***P*< 0.01, ****P*< 0.001.
